# Fish oil reverses metabolic syndrome, adipocyte dysfunction, and altered adipokines secretion triggered by high‐fat diet‐induced obesity

**DOI:** 10.14814/phy2.14380

**Published:** 2020-02-28

**Authors:** Roberta D. C. da Cunha de Sá, Maysa M. Cruz, Talita M. de Farias, Viviane S. da Silva, Jussara de Jesus Simão, Monica M. Telles, Maria Isabel C. Alonso-Vale

**Affiliations:** ^1^ Post‐graduate Program in Chemical Biology Institute of Environmental Sciences, Chemical and Pharmaceutical Federal University of Sao Paulo ‐UNIFESP Diadema Sao Paulo Brazil; ^2^ Department of Biological Sciences Institute of Environmental Sciences, Chemical and Pharmaceutical Federal University of Sao Paulo ‐ UNIFESP Diadema Sao Paulo Brazil

**Keywords:** adipose tissue, cytokines, dyslipidemia, inflammation, insulin resistance, omega‐3 fatty acids

## Abstract

The effect of fish oil (FO) treatment on high‐fat (HF) diet‐induced obesity and metabolic syndrome was addressed by analyzing dysfunctions in cells of different adipose depots. For this purpose, mice were initially induced to obesity for 8 weeks following a treatment with FO containing high concentration of EPA compared to DHA (5:1), for additional 8 weeks (by gavage, 3 times per week). Despite the higher fat intake, the HF group showed lower food intake but higher body weight, glucose intolerance and insulin resistance, significant dyslipidemia and increased liver, subcutaneous (inguinal‐ING) and visceral (retroperitoneal‐RP) adipose depots mass, accompanied by adipocyte hypertrophy and decreased cellularity in both adipose tissue depots. FO treatment reversed all these effects, as well as it improved the metabolic activities of isolated adipocytes, such as glucose uptake and lipolysis in both depots, and de novo synthesis of fatty acids in ING adipocytes. HF diet also significantly increased both the pro and anti‐inflammatory cytokines expression by adipocytes, while HF + FO did not differ from control group. Collectively, these data show that the concomitant administration of FO with the HF diet is able to revert metabolic changes triggered by the diet‐induced obesity, as well as to promote beneficial alterations in adipose cell activities. The main mechanism underlying all systemic effects involves direct and differential effects on ING and RP adipocytes.

## Graphical abstract

The manuscript show relevant effects of fish oil (FO) treatment reversing both the metabolic syndrome induced by high‐fat diet (HF) and the changes in subcutaneous (inguinal ‐ING) and visceral (retroperitoneal ‐RP) adipocytes, such as glucose uptake, lipolysis, and lipogenesis. The major findings of our study is that the FO treatment reverses the deleterious effects caused by the excessive intake of a HF diet, and the mechanism involves, at least in part, direct and differential effects on ING and RP adipocytes. Altogether, these effects prevented adipocyte hypertrophy caused by HF diet and reflected in decreased adiposity. Additionally, FO treatment decreased the gene and protein expression of inflammation‐related factors. Thus, the work provided important evidence concerning the effectiveness of a low‐cost agent (as the FO) to treat the metabolic and inflammatory disorders triggered by obesity, which causes thousands of deaths annually and burdens the public coffers.

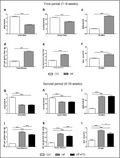

## 1 INTRODUCTION

Overweight and obesity are defined as abnormal or excessive fat accumulation characterized by energy imbalance between consumption and calorie expenditure and presents a potential risk to the health (Manzel et al., 2014; Mittwede, Clemmer, Bergin, & Xiang, 2016). Diet quality is described as one of the main factors associated with appearance of metabolic syndrome (MS) components such as hyperglycemia, hypertriglyceridemia, hypertension, decreased high‐density lipoproteins (HDL) cholesterol and increased waist circumference, that triggers obesity‐related comorbidities.

The white adipose tissue (WAT) can be found in several regions, such as subcutaneous region, under the extension of the skin, or deeper regions, connecting to the viscera. Fat depots in different areas of the body present distinct structural and functional properties (lipolysis, lipogenesis, glucose uptake, production and secretion of hormones and cytokines), as well as different responses to external stimulus (diets, exercises, drugs, among others) (Berg & Scherer, 2005; Kranendonk et al., 2015). Obesity results in WAT dysfunction characterized by hypertrophied adipocytes, altered immune cell profile present in the stromal and dysregulated cytokine expression and secretion (Hajer, Haeften, & Visseren, 2008; Weisberg et al., 2003).

Administration of a high‐fat (HF) diet in rodents is a well‐known experimental model for raising body mass gain and promoting fat accumulation (Ramalho, Jornada, Antunes, & Hidalgo, 2017), whereas fish oil (FO) supplementation has been shown to reduce body fat in these animals (Rokling‐Andersen et al., 2009). FO is rich in long‐chain omega‐3 polyunsaturated fatty acids (LC *n* − 3 PUFAs). LC *n* − 3 PUFAs, including eicosapentaenoic acid (EPA 20:5) and docosahexaenoic acid (DHA 22:6), have long been shown to improve insulin sensitivity and to play a potent anti‐inflammatory, hypolipidemic, and body weight (BW) reducing effects (Chen, Xu, Yan, Yu, & Li, 2015; Couet, Delarue, Ritz, Antoine, & Lamisse, 1997; Kopecky et al., 2009; de Sá et al., 2016; Serhan, 2014). The most important source of EPA and DHA is marine fish, such as salmon, sardines, tuna, and mullet (Rustan, Hustvedt, & Drevon, 1993).

Due to the concern about obesity and its metabolic consequences, and the recognition that adipocytes comprise a wide range of homeostatic processes, there is interest in the study of metabolic and endocrine function of WAT. We have previously described that FO presents beneficial effects on preventing obesity and MS, due in part to the effects on depot‐specific isolated adipocytes, since previous FO administration (initiated 4 weeks before the induction of obesity) prevented inguinal (ING) and retroperitoneal (RP) adipocytes dysfunction induced by the HF diet (de Sá et al., 2016). Herein, we investigated if FO displays the same beneficial effects on already dysfunctional adipose cells triggered by obesity. In order to address this question, we elaborated a protocol to firstly induce obesity by HF diet in mice and after that, to evaluate the treatment with FO.

The information obtained in this study might contribute to the elucidation of metabolic and endocrine dysfunctions (regional) of isolated adipocytes from different adipose depots that occur in obesity as well as the potential therapeutical use of LC *n* − 3 PUFAs on treating these cells. The major findings of our work is that FO treatment, containing high concentration of EPA compared to DHA (5:1), 3 times per week, reverses the deleterious effects caused by the excessive intake of a HF diet. The mechanism of action involves direct and differential effects on ING and RP adipocytes.

## 2 MATERIAL AND METHODS

### 2.1 Ethical approval

The study was performed according to protocols approved by the Ethics Committee of the Federal University of São Paulo (CEUA 7912040515).

### 2.2 Animals and fish oil supplementation

Eight‐week‐old male C57BL/6 mice obtained from the Center for Development of Experimental Models (CEDEME), Federal University of São Paulo (UNIFESP), were housed in a room with light‐dark cycle of 12–12 hr and temperature of 24 ± 1°C. The experimental protocol remained for 16 weeks, consisted in the first 8 weeks (first period) to divide mice into two groups: control (CO, 9% fat, 76% carbohydrates, and 15% proteins) and HF diet (HF, 26% carbohydrates, 59% fat, and 15% proteins) group. In the next 8 weeks (second period), the HF group was subdivided into HF and HF + FO (HF diet supplemented with FO) groups. Supplementation was performed three times per week, by oral gavage at 2 g/kg BW (LC *n* − 3 PUFA source, 5:1 EPA/DHA ratio, HiOmega‐3, Naturalis Nutrição and Farma Ltda). The CO and HF groups received water by gavage at the same volume (~50 µl, according to the BW). The FO dosage was chosen based on previous studies from our group (Amaral et al., 2015; Reagan‐Shaw, Nihal, & Ahmad, 2008; de Sá et al., 2016).

### 2.3 Experimental procedure

Body weight and food intake were measured once a week. After 16 weeks of the experimental protocol, 6 hr fasted mice were anesthetized with isoflurane and killed by cervical dislocation after blood collection through puncturing the orbital plexus. Blood samples were centrifuged at 1,200 *g* for 20 min at 4°C and serum was stored at −80°C. Adipose fat depots (ING and RP) were harvested, weighed, and processed as described below.

### 2.4 Glucose and insulin tolerance test

Tolerance to glucose (GTT) or to insulin (ITT) was performed in the last week of the treatment protocol. Mice were fasted for 6 hr and injected intraperitoneally with glucose (2 g/kg BW) to GTT, or insulin (0.75 UI/kg BW; Humulin R; Lilly) to ITT. Tail‐vein blood glucose levels were determined before and 15, 30, 45, 60, and 90 min after glucose injection, and before and 3, 6, 9, 12, and 15 min after insulin injection using a OneTouch^®^ glucometer (Johnson & Johnson). Glucose concentration versus time was plotted and the area under the curve was calculated for each animal, and glucose concentration versus time was plotted and the glucose level lowering rate was calculated.

### 2.5 Blood measurements

Triacylglycerol (Bucolo & David, 1973), total cholesterol, LDL‐cholesterol (Postiglione et al., 1982), and HDL‐cholesterol levels (Grub, Mazzotti, & Murador, 1981) were determined by colorimetric assays (Labtest Diagnostics).

### 2.6 Adipocyte isolation

Adipocyte isolation was performed as described previously (Rodbell, 1964) with slight modifications (de Sá et al., 2016). A small number of adipocytes were photographed under an optical microscope (100× magnification) using a microscope camera (Moticam 1000; Motic) and mean adipocyte diameter was determined by measuring 50 cells using Motic‐Images Plus 2.0 software.

### 2.7 Lipolysis determination

Lipolysis was estimated as the rate of glycerol [Free Glycerol Determination Kit, Sigma Sigma) (Wellman & Wilson, 1962) released from ING and RP adipocytes (1 × 10^6^/cells) during 30 min of incubation under basal or stimulated [by isoproterenol (2 × 10^–6^ M)] conditions. Similar procedure was used by Bolsoni‐Lopes et al. (2013). Results are expressed as nmol of glycerol per 1 × 10^6^ adipocytes.

### 2.8 Incorporation of [^3^H]‐oleate into triacylglycerol

ING and RP adipocytes (10^6^ cells/ml) were incubated in Krebs/Ringer/phosphate buffer (pH 7.4) containing BSA (1%), glucose (2 mM) and [3H]‐oleate (100 μM, 1850 Bq per tube or well) for 2 hr at 37°C in a water bath. At the end of the incubation period, the mixture was transferred to a 1.5 ml tube containing 400 μl of silicone oil and centrifuged for 30 s. The cell pellet on the top of the oil layer was transferred to polypropylene tubes containing 2.5 ml of Dole's reagent for lipid extraction. After addition of *n*‐heptane (1.5 ml) and distilled water (1.5 ml), tubes were vortexed and the mixture decanted for 5min. An aliquot of the upper phase was collected into a scintillation vial for determination of radioactivity trapped into TAG (1450 LSC, CouterMicro‐Beta, Trilux; Perkin Elmer). Results are expressed as nmol of oleate incorporated into TAG per 1 × 10^6^ cells/h. A similar procedure was used in our previous study (Bolsoni‐Lopes et al., 2013).

### 2.9 Incorporation of [1‐^14^C]‐acetate into fatty acids

ING and RP adipocytes (10^6^ cells/ml) were incubated in Krebs/Ringer/phosphate buffer (pH 7.4) containing BSA (1%), glucose (2 mM) and [1‐14C]‐acetate (1 mM, 1850 Bq per tube or well) for 2 hr at 37°C in a water bath. The results are expressed as nmol acetate incorporated into TAG per 1 × 10^6^ cells/h. A similar procedure was described in our previous study (Bolsoni‐Lopes et al., 2014).

### 2.10 2‐Deoxy‐D‐glucose (2‐DG) uptake

ING and RP adipocytes (1 × 10^6^ cells/ml) were incubated with or without insulin (10 nmol/L) in buffer composed of (mM): 140 NaCl, 20 Hepes, 5 KCl, 2.5 MgSO_4_, 1 CaCl_2_, and BSA 1% (pH 7.4) for 20 min at 37°C. Subsequently, 2‐deoxy‐D‐[3H]‐glucose (0.4 mmol/l, 1850 Bq per tube or well) was added and the reaction was allowed to occur for exactly 3 min. The reaction was interrupted by adding 250 μl of ice‐cold phloretin (0.3 mmol/L in Earle's salts, Hepes 10 mm, BSA 1%, and DMSO 0.05%). At the end of incubation, the glucose uptake was measured as described in our previous studies (de Sá et al., 2016). The results are expressed as pmol per 1 × 10^5^ adipocytes.

### 2.11 RNA extraction and quantitative real‐time polymerase chain reaction (qPCR)

Total RNA from ING and RP isolated adipocytes was extracted using Trizol (Invitrogen Life Technologies), analyzed for quality on agarose gel and absorbance ratios of 260/280 and 260/230 nm, and reverse transcribed to cDNA using the SuperScript III cDNA kit (Invitrogen Life Technologies). Gene expression was evaluated by real‐time qRT‐PCR using a Rotor Gene (Qiagen) and SYBR Green as fluorescent dye (Qiagen). The reaction conditions were: 95°C for 5 min, then 40 cycles of 95°C for 5 s and 60°C for 10 s. PCR products were run on agarose gel to confirm the size of the fragment and specificity of amplification. The primers used are: 36B4 (5′‐3′ sense: TAAAGACTGGAGACAAGGTG; 5′‐3′ antisense: GTGTACTCAGTCTCCACAGA), adiponectin (5′‐3′ sense: GCAGAGATGGCACTCCTGGA; 5′‐3′antisense: CCCTTCAGCTCCTGTCATTCC), CEBP‐α (5′‐3′ sense: CGCAAGAGCCGAGATAAAGC; 5′‐3′antisense: CAGTTCACGGCTCAGCTGTTC), IL‐10 (5′‐3′ sense: CTGGACAACATACTGCTAACCG; 5′‐3′ antisense: GGGCATCACTTCTACCAGGTAA), IL‐6 (5′‐3′ sense: TTCTCTGGGAAATCGTGGAAA; 5′‐3′ antisense: TCAGAATTGCCATTGCACAAC), leptin (5′‐3′ sense: CATCTGCTGGCCTTCTCCAA; 5′‐3′ antisense: ATCCAGGCTCTCTGGCTTCTG), MCP‐1 (5′‐3′ sense: GCCCCACTCACCTGCTGCTACT; 5′‐3′ antisense: CCTGCTGCTGGTGATCCTCTTGT), perilipin (5′‐3′ sense: AGTGTGGGGTCCTTGGGCGT; 5′‐3′ antisense: TGGCAGCTGTGAACTGGGTGG), PPAR‐γ (5′‐3′ sense: GCATCAGGCTTCCACTATGGA; 5′‐3′ antisense: AAGGCACTTCTGAAACCGACA), resistina (5′‐3′ sense: AGACTGCTGTGCCTTCTGGG; 5'‐3' antisense: CCCTCCTTTTCCTTTTCTTCCTTG), TNF‐α (5′‐3′ sense: CCCTCACACTCAGATCATCTTCT; 5′‐3′ antisense: GCTACGACGTGGGCTACAG). 36B4 was used as housekeeping. The results were obtained as Ct values (Ct = cycle number at which logarithmic PCR plots cross a calculated threshold line) and used to determine ΔCt values [ΔCt = (Ct of the target gene) − (Ct of the housekeeping gene)]. The results are expressed as arbitrary units using the transformation: expression = 1,000 × (2^−∆ct^) arbitrary units.

### 2.12 Adipokine measurements

Isolated adipocytes (~8 × 10^5^/cells) were incubated in a six‐well culture plate in Dulbecco's modified Eagle's medium containing 10% calf serum and 1% penicillin/streptozotocin at 37°C under humid atmosphere of 5% CO_2_ for 30 hr. The final concentrations of the adipokines adiponectin, IL‐10, IL‐6, MCP‐1, resistin, and TNF‐α from the culture supernatant were determined using specific commercially available DuoSet ELISA kits in accordance with the manufacturer's (R&D Systems; catalogue numbers DY1119, DY417, DY406, DY479, DY1069 and DY 410, respectively). The concentrations of the cytokines are expressed in ng per 10^6^ cells as indicated. A similar procedure was used in our previous studies (de Sá et al., 2016).

### 2.13 Statistical analysis

Data are expressed as mean ± standard error of the mean (*SEM*). Student's *t* test was used in the first period, while one‐way ANOVA variance analysis, followed by Tukey's post‐test, were used in the second period for comparisons between groups. Prism, version 5.0 (GraphPad Software, Inc.) was used for analysis. *p* *<* .05 was considered statistically significant.

## 3 RESULTS

### 3.1 Food ingestion and body weight gain

**Figure 1:**
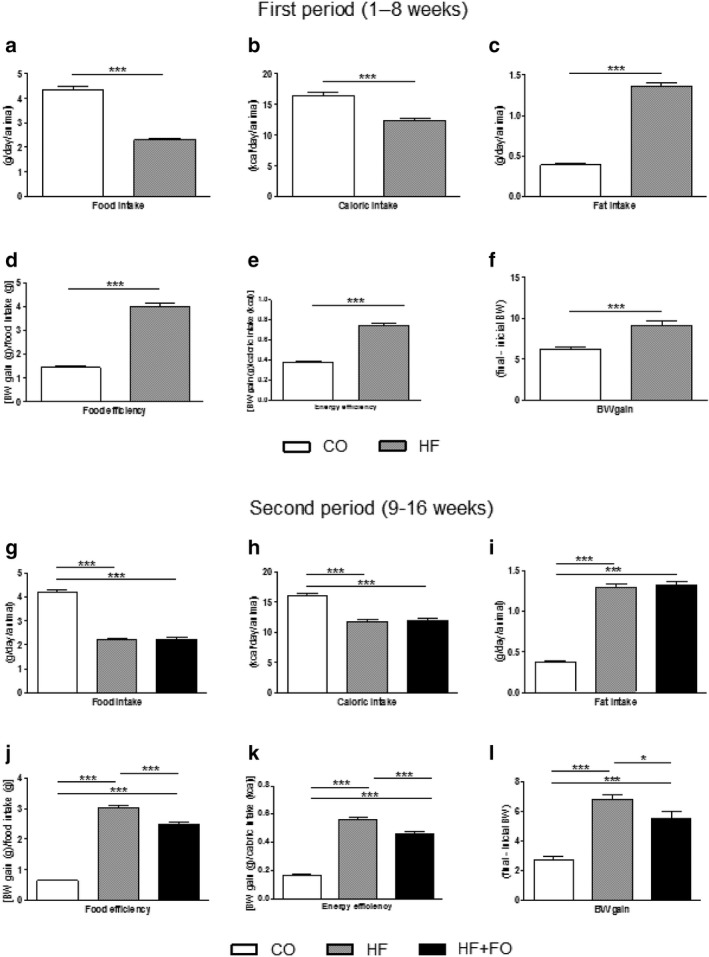
Food intake, energy efficiency, and body weight (BW) gain of mice fed with control (CO) or high‐fat (HF) diet and treated with fish oil (FO). In the first period, the animals received CO or HF diet. In the second period, the diets were maintained and the animals received water (CO and HF groups) or fish oil (HF + FO group) by oral gavage. Food intake (a), caloric intake (b), fat intake (c), food efficiency (d), energy efficiency (e) and BW gain (f) in the first period. Food intake (g), caloric intake (h), fat intake (i), food efficiency (j), energy efficiency (k), and BW gain (l) in the second period. Mean ± *SEM* (*n* = 10–20). *p < .05, ***p < .001

In the first period of experimental protocol (1–8 weeks), while animals received only CO or HF diets without FO treatment, mice fed with the HF diet presented a decrease in food (by 46%; *p* < .05) and caloric intake (25%; *p* < .05) compared to the CO animals (Figure 1a and b). The HF group presented an increase in fat intake, food, and energy efficiency (by 2,5‐, 3‐, and 2‐fold, respectively; *p* < .05) (Figure 1c–e). The mean of BW at the beginning of the protocol was 21 g while at the end of the first period, the CO group presented a BW mean increase of 6.10 g, while the HF group presented 9.1 g of BW (47% more than the CO group; *p* < .05) (Figure 1f).

In the second period (9–16 weeks), similar results were observed concerning food, caloric and fat intake comparing HF and HF + FO groups (Figure 1g–i). However, food and energy efficiency remained higher in HF, but not in HF‐FO group, since a partial reduction in both parameters was observed (by 17%; *p* < .05, Figure 1j and k). About the BW mass, the animals from the HF group had an 152% increase in body mass (gain of 6.8 g) when compared to the CO group (gain of 2.7 g; *p* < .05). The HF + FO group was still 104% higher in relation to CO group (gain of 5.5 g), but presented a significant reduction of ~ 19% (*p* < .05) compared to the HF group, as illustrated in Figure 1l.

### 3.2 Glucose and insulin tolerance tests, plasma and hepatic lipid profile

**Figure 2:**
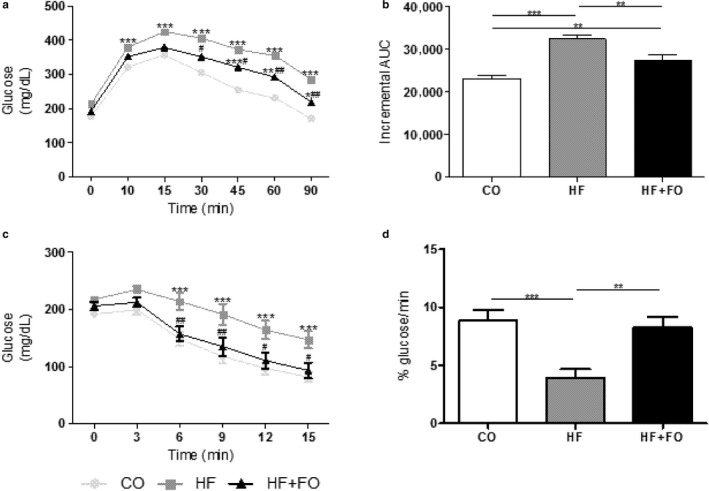
GTT and ITT of mice fed with CO or HF diet and treated with FO*.* (a) GTT or glucose concentration versus time after administration of glucose. (b) incremental area under the curve from GTT. (c) ITT or glucose decay curve versus time after insulin administration. (d) glucose decrease rate for ITT (kITT). Mean ± *SEM* (*n* = 8–16). *p < .05, **p < .01, ***p < .001 versus CO diet. *^#^p* < .05, *^##^p* < .01 versus HF diet

Compared to the CO diet, mice fed with HF diet showed glucose intolerance and a significant increase in the area under the curve by 40% (Figure 2a and b; *p* < .05), as well as insulin intolerance and a lower glucose disappearance rate by 56% (Figure 2c and d; *p* < .05). The treatment with FO partially reversed the response to glucose overload (glucose intolerance) triggered by the HF diet while it totally restored the insulin sensitivity.

The consumption of a HF diet for 16 weeks resulted in an increase of 80% (*p* < .05) in serum concentrations of triglyceride, 26% (*p* < .05) on total cholesterol and 37% (*p* < .05) on LDL cholesterol as well as on absolute liver weight gain (12%; *p* < .05) and concentration of triglycerides in this organ (77%; *p* < .05) when compared to the CO group. The treatment with FO during the last 8 weeks completely reversed these parameters. In this same group (HF + FO), HDL cholesterol showed a higher plasma concentration when compared to CO (19%; *p* < .05) (Table 1).

**Table 1:** Plasma measurements in mice fed with CO or HF diet and treated with FO

	CO	HF	HF + FO
Triglycerides (mg/dl)	80.19 ± 4.42	144.48 ± 14,00***	102.21 ± 6.30^##^
Total cholesterol (mg/dl)	174.34 ± 6.82	220.22 ± 14.50**	175.96 ± 7.53^##^
LDL cholesterol (mg/dl)	59.64 ± 3.33	81.66 ± 4.36**	64.53 ± 5.56^#^
HDL cholesterol (mg/dl)	84.37 ± 2.48	96.03 ± 4.69	100.79 ± 4.75*
Liver (g)	0.98 ± 0.02	1.10 ± 0.04*	0.99 ± 0.03^#^
Triglycerides in the liver (mg/dl)	70.71 ± 11.96	125.25 ± 17.61*	62.40 ± 5.15^##^

Note

In the first period, the animals received control (CO) or high‐fat (HF) diet. In the second period, the diets were maintained and the animals received water (CO and HF groups) or fish oil (HF + FO group) by oral gavage. Mean ± *SEM* (*n* = 10–20). *p < .05, **p < .01, ***p < .001 versus CO diet. *^#^p* < .05, *^##^p* < .01 versus HF diet.

### 3.3 Adiposity, adipocytes volume, cellularity, and gene expression of adipogenic markers

**Figure 3:**
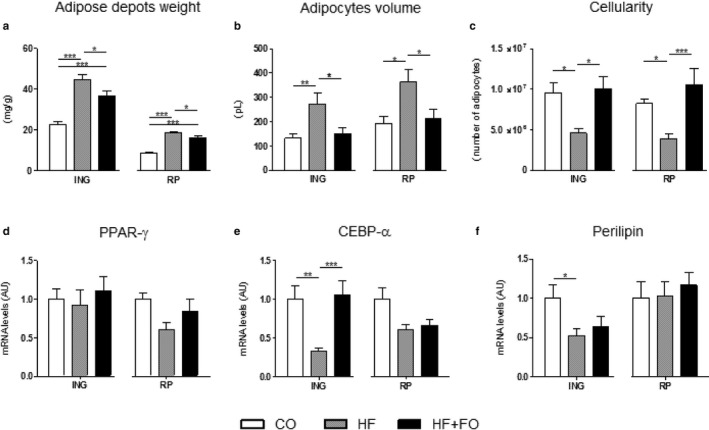
Adiposity, hypertrophy of adipocytes, cellularity, and mRNA levels of adipogenic markers from ING and RP fat depots of mice fed with CO or HF diet and treated with FO*.* (a) relative weight (mg/g BW) of ING and RP fat depot. (b) ING and RP volume adipocytes. (c) ING and RP cellularity. mRNA levels of PPAR‐γ (d), CEBP‐α (e) and perilipin (f) in ING and RP adipocytes. 36B4 was used as the housekeeping gene. Mean ± *SEM* (*n* = 10–19). *p < .05, **p < .01, ***p < .001

After euthanasia, the ING and RP adipose depots were removed and weighed. Statistical analysis showed that HF diet for 16 weeks promoted an expressive increase in the mass of these depots (96% and 113%, respectively), compared to the CO group. FO treatment during the last 8 weeks of protocol partially reversed this effect by 17% and 13%, in both ING and RP adipose depots, respectively (Figure 3a; *p* < .05).

Analyzing the isolated cells from these adipose depots, the HF group presented ING and RP adipocytes hypertrophy by 109% and 86%, respectively, when compared to the CO, whereas the FO treatment resulted in a reduction by 45% and 41%, respectively, presenting similar values to the CO group (Figure 3b; *p* < .05).

For both tissues, HF group presented lower number of adipocytes when compared to their respective CO. The treatment with FO concomitant with the HF diet increased the number of adipocytes by 119% in the ING depot and 170% in the RP depot, compared to the HF group (Figure 3c; *p* < .05).

C/EBPα and PPARγ are both required to adipogenesis and to maintain the differentiated state of mature adipocytes and insulin sensitivity. They activate its target genes such as perilipin, that together, represents important markers of adipocyte differentiation. We observed a significant decrease in ING adipocytes for the expression of *Cebpa* and *Perilipin* (of 67% and 47%, respectively; Figure 3e and f) in the HF group when compared to the CO, corroborating the decrease in the cellularity seen in the same group. The *Cebpa* expression was completely reversed by FO treatment. No statistical difference was observed for *Ppparg* (Figure 3d).

### 3.4 Metabolic activities of isolated adipocytes

In order to investigate whether FO treatment influence the glucose and TAG metabolism in adipocytes from obese mice induced by HF diet, glucose uptake, lipogenesis and lipolysis tests were performed in these cells.

**Figure 4:**
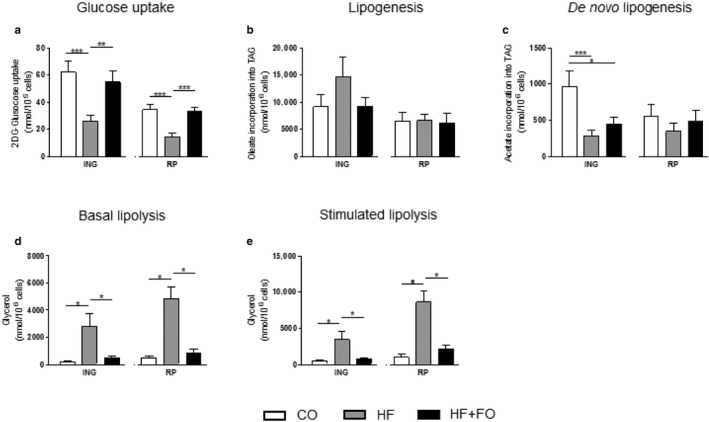
Metabolic activities in isolated adipocytes from ING and RP fat depots of mice fed with CO or HF diet and treated with FO. Basal 2DG‐glucose uptake (a), oleate incorporation into TAG (b), acetate incorporation into TAG (c), basal lipolysis (d) and isoproterenol (e) stimulated lipolysis by ING and RP adipocytes. Mean ± *SEM* (*n* = 8–15). *p < .05, **p < .01, ***p < .001

HF diet induced a significant reduction (by ~57%) in glucose uptake by both ING and RP adipocytes, in comparison to their respective CO. The group receiving FO treatment in both cells showed a total reversion of this effect, showing approximately a 2‐fold increment on glucose uptake (*p* < .05) in comparison to the HF group (Figure 4a).

We next investigated whether FO also affects lipid metabolism. No statistical significance was reached in relation to the incorporation of oleate into TAG in ING and RP adipocytes (Figure 4b). The de novo synthesis of FA from acetate in the RP depot was also similar among the three groups but a significant reduction was observed in ING adipocytes from the HF and HF + FO groups (by 71% and 54%, respectively; Figure 4c; *p* < .05).

Cells were finally evaluated for lipolysis activity. The HF diet resulted in a higher rate of lipolysis at the basal (12.8‐fold and 9.5‐fold; *p* < .05) and stimulated (6,5‐fold and 8,7‐fold; *p* < .05) conditions in ING and RP adipocytes, respectively, when compared to their respective CO groups. The HF + FO group presented a reduction of ~80% in both cells at basal conditions, and of 79% and 75% at stimulated conditions for ING and RP adipocytes, respectively (Figure 4d and e; *p* < .05).

### 3.5 Gene expression and secretion of adipokine by isolated adipocytes

**Figure 5:**
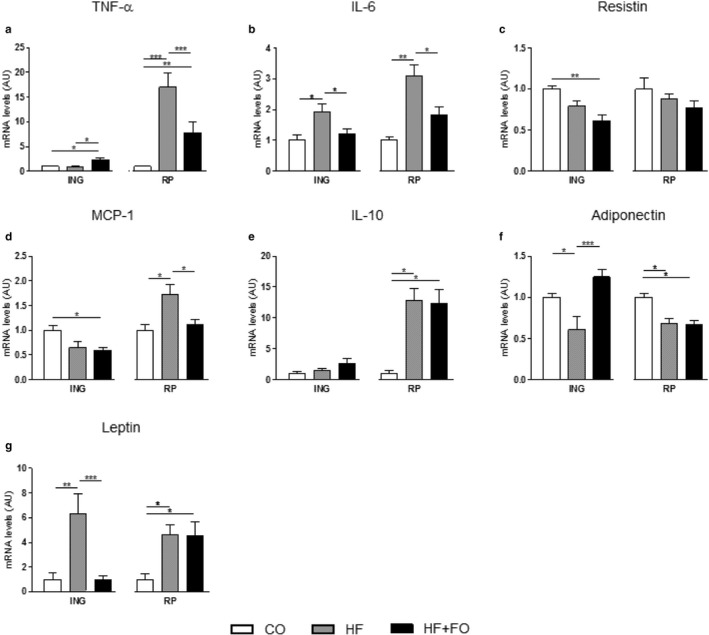
mRNA levels and secretion of cytokines expressed by isolated adipocytes from ING and RP fat depots of mice fed with CO or HF diet and treated with FO. mRNA levels of TNF‐*α* (a), IL‐6 (b), resistin (c), MCP‐1 (d), IL‐10 (e), adiponectin (f) and leptin (g) in ING and RP adipocytes. 36B4 was used as the housekeeping gene. Mean ± *SEM* (*n* = 8–10). *p < .05, **p < .01, ***p < .001

**Figure 6:**
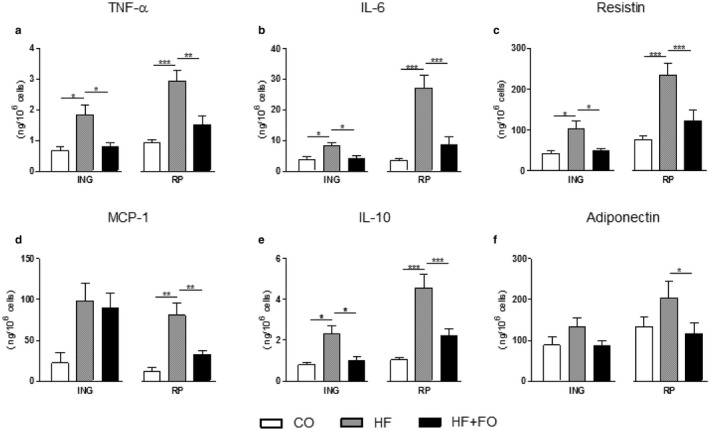
Secretion of cytokines by isolated adipocytes from ING and RP fat depots of mice fed with CO or HF diet and treated with FO. Secretion of TNF‐*α* (a), IL‐6 (b), resistin (c), MCP‐1 (d), IL‐10 (e), and adiponectin (f) by ING and RP isolated adipocytes. Mean ± *SEM* (*n* = 8–10). *p < .05, **p < .01, ***p < .001

The expression of genes encoding important cytokines involved in inflammation was measured in isolated adipocytes from both ING and RP depots. The *Tnfa* gene expression in RP adipocytes showed a significant increase (17‐fold; *p* < .05) in HF group which was totally reversed by FO treatment, reaching a reduction of 54% (*p* < .05; Figure 5a). The expression of *Il6* was increased in response to HF diet in both depots ING and RP (by 90% and 210%, respectively; *p* < .05), but reversed by FO treatment (Figure 5b). The *Resistin* gene expression was reduced by 40% (*p* < .05) only in the ING adipocytes of HF + FO group when compared to the CO (Figure 5c). The *Mcp1* expression presented difference only in the RP adipocytes, where the HF group showed an increase of 70% (*p* < .05) compared to the CO group; the treatment with FO reversed this effect with a reduction of 35% (*p* < .05) in the HF + FO group as compared to HF (Figure 5c and d). The *Il10* gene expression was shown to be higher expressed (12.5‐fold; *p* < .05) in both HF and HF + FO groups compared to the CO group, only in RP adipocytes (Figure 5e). Regarding *Adipoq* expression, Figure 6f shows a reduction of 40% (*p* < .05) in the HF group compared to CO group, and an increase of 200% (*p* < .05) in the HF + FO group when compared to the HF in ING adipocytes. Regarding to RP adipocytes, there was a reduction in both groups receiving HF diet (30%; Figure 5f). The expression of *Leptin* was significantly higher in both HF and HF + FO groups (4.5‐fold; *p* < .05) compared to CO in RP adipocytes and in the HF group compared to CO (6.3‐fold; *p* < .05) in ING adipocytes; however, in the latter, FO treatment reversed the *Leptin* increase (Figure 5g).

We also evaluated the cytokines secretion by isolated adipocytes. Reinforcing gene expression data, we observed a significant increase of TNF‐α, IL‐6, resistin and IL‐10 in HF group compared to CO in both ING (2.6, 2.2, 2.5, and 2.9‐fold; *p* < .05) and RP (3.2, 7.8, 3.0, and 4.6‐fold; *p* < .05) adipocytes, respectively. The FO treatment was able to completely reverse this effect in both depots (Figure 6a, b, c and e; *p* < .05). The secretion of MCP‐1 showed a significant increase only in the RP adipocytes, being 7.1 fold higher in the HF group compared to the CO; however, FO treatment caused a reduction of 60% on MCP‐1 secretion in HF + FO compared to the HF group (Figure 6d; *p* < .05). No statistical difference was observed in adiponectin secretion in ING adipocytes, but in RP adipocytes it can be seen a reduction in HF + FO group (by 42%; *p* < .05) compared to HF group (Figure 6f).

## 4 DISCUSSION

We investigated the effect of FO on treating mice with HF diet‐induced obesity, insulin resistance and dysfunctional adipocytes. Our main results show that, even with a partial reduction in body mass, the FO administration simultaneously with the continuous intake of HF diet was able to reverse glucose intolerance, insulin resistance and dyslipidemia, as well as to reduce adipocyte hypertrophy and reestablish its functions, such as endocrine (cytokines secretion) and metabolic (glucose uptake, lipolysis and lipogenesis) in white adipose depots from inguinal and retroperitoneal regions. Here, we emphasize the very substantial effect of FO in modulating pro‐inflammatory and anti‐inflammatory adipokines expression and secretion by isolated adipocytes.

These reversal effects occurred in the absence of changes in dietary intake such as lipid and caloric intake, since both HF and HF + FO groups consumed similar food, calories and fat amounts. Corroborating these findings, previous studies have shown that eating EPA‐enriched high‐fat diets reduced diet‐induced obesity and insulin resistance in rodents without affecting energy intake (LeMieux, Kalupahana, Scoggin, & Moustaid‐Moussa, 2015; Oliveira et al., 2019).

Comparing the diets CO and HF, it can be seen that even eating less food and calories, poor distribution of macronutrients due to the HF intake potentially influences not only the increase in body mass but also all the negative metabolic consequences associated with obesity, such as glucose intolerance and insulin resistance and dyslipidemia. It is known that this condition might lead to the development of cardiovascular disease – CVDs, wherein inadequate fat intake causes 17 million deaths per year, according to World Health Organization (Mittwede et al., 2016).

Although in our protocol, mice fed a HF diet for 16 weeks after receiving FO treatment (during weeks 9–16) did not present any difference in food and caloric intake compared to HF group, the treatment was able to reduce, even though in part, the body mass of the animals, as well as food and energy efficiency.

Following the increase in body mass, the HF diet also induced MS in mice, corroborating our previous published work demonstrating that this duration and composition of HF diet results in obesity, glucose, and insulin intolerance, increased fasting blood glucose and insulin levels, raised blood cholesterol and low density lipoproteins (LDL) cholesterol concentrations, and elevated homeostatic model assessment of insulin resistance (HOMA‐IR), thus characterizing the MS (de Sá et al., 2016). In addition to the reduction in glucose intolerance, insulin resistance, and dyslipidemia, FO treatment completely reversed the mass and lipid content of the liver. Importantly, dyslipidemia is often observed in obese and/or diabetic individuals and high plasma concentrations of total and LDL cholesterol are associated with an increased risk of CVD (Burdge & Calder, 2015; Zalesin, Franklin, Miller, Peterson, & Mccullough, 2011).

WAT has multiple depots that exhibit distinct functional properties. They are distributed through several regions, such as the subcutaneous region, under the extension of the skin, or deeper regions, connecting to the viscera. In this study, the effect of the HF diet on two different WAT was studied. Both ING subcutaneous and the visceral RP adipose depots of the HF diet‐induced obese animals presented an increase in their mass (96% and 113.6%, respectively), and both also had their cells (adipocytes) hypertrophied when compared to the CO group (109% and 86%, respectively).

It is known that ω‐3 FA is able to reduce hypertrophy of adipocytes (Kopecky et al., 2009; de Sá et al., 2016) as well as the growth of adipose mass, which many authors justify it by an inhibition of adipocytes proliferation (Adamcova et al., 2018; Hensler et al., 2011; Masoodi, Kuda, Rossmeisl, Flachs, & Kopecky, 2014). Interestingly, we observed that the treatment with ω‐3 partially reduced the WAT mass depot (17% in ING and 13% in RP) and when the size of the cells was measured, there was a total reversal of hypertrophy with a concomitant increase in hyperplasia in both depots (119% and 170% of increase in cell number for ING and RP, respectively). We also observed an increase in gene expression of two important adipogenic markers (*Cebpa* and *Perilipin)* in ING depot corroborating the higher number of cells in HF + FO group, which justifies the fact that these depots are not hypertrophied but still lead up to a tissue with a larger mass compared to CO. Taking together, it can be concluded that FO, rich in EPA compared to DHA (5:1), was highly effective in reversing the hypertrophy triggered by obesity, independent of exerting only a partial reduction in adipose mass, or even an increment in the adipose depot cells amount.

Obesity is mainly mediated by changes in size (hypertrophy by increased cell volume) of mature adipocytes that may occur in response to the activation of its typical metabolic actions, lipogenesis, and lipolysis. These functions vary with the need for lipids incorporation or release, which depend, among other factors, on the individual's nutritional status, energy expenditure, the influence of hormones (catabolic or anabolic), the activity of enzymes involved in these processes and characteristic heterogeneity among the various adipose groups of the organism (Jensen, 1997). Interestingly, unlike the results we published previously (de Sá et al., 2016) in which after 8 weeks (and not 16) of HF diet administration, lipogenesis was exacerbated in the HF group and prevented by FO supplementation (initiated 4 weeks prior to the introduction of the HF diet); here, we did not observe any significant changes in lipogenesis (only a tendency to increase in adipocytes from the HF group. We postulate that the long period of lipid supply by the HF diet is somewhat compensated by an increase in the quantity of smaller and younger adipocytes in FO treated animals, able to uptake and metabolize more efficiently the excess of lipids from the circulation, preventing the lipotoxicity in other tissues as observed in this study by the decrease of TAG in liver. The presence of smaller and more numerous adipocytes is now accepted to correlate with healthy adipose tissue expansion, preventing the development of hypertrophic obesity, an independent risk factor for the development of type 2 diabetes (Gustafson, Hammarstedt, Hedjazifar, & Smith, 2013).

Concerning the de novo FA synthesis, we observed a significant reduction in both groups that received the HF diet in this metabolic pathway, although significant only in ING adipocytes. This reduction was already expected since the cells were exposed to an abundance of lipid‐rich dietary FA, and there is no need for de novo synthesis (Delgado et al., 2009; Gaidhu, Anthony, Patel, Hawke, & Ceddia, 2010; de Sá et al., 2016).

We also evaluated the lipolytic activity of the adipocytes. Comparing the tissues, looking at CO animals, the RP depot was found to be more lipolytic, corroborating the data in literature (Berg & Scherer, 2005; Kranendonk et al., 2015). We also observed that adipocytes from HF diet‐induced obese animals, which showed hypertrophied, presented increased lipolysis when compared to ones from CO group in both depots, although higher in visceral (RP) depot. FO treatment promoted a total reversion of this effect, reaching similar values to the CO group. It is known that elevated levels of FFA are associated to higher deposition of these lipids in other organs and, more aggressively, it is correlated to central adiposity (associated with the viscera), which corroborates for fat accumulation in heart and liver (lipotoxic effects) (Chen et al., 2015; Su, Lee, Cheng, & Huang, 2015).

Although ING tissue is not so lipolytic as RP, considered a FFA‐releasing tissue, it actually contributes to the worsening of hyperglycemia and insulin resistance in HF diet group since the ability to uptake glucose is impaired in ING adipocytes from these animals [13]. In the present study, CO ING cells presented approximately twice as much glucose uptake as HF ING cells, which, considering their greater amount in the body, justifies their greater and positive correlation with glycemic homeostasis. Although RP adipocytes present lower glucose uptake compared to ING, they also directly contribute to the increase in glycemia since, among other factors, their higher lipolytic response results in a greater release of FFA, which impairs the expression and recruitment of GLUT‐4 glucose transporters to the cell surface in insulin‐sensitive tissues. In both, ING and RP isolated adipocytes, the decrease in glucose uptake triggered by HF diet intake was reversed by FO treatment. Similar to the present results, studies with EPA and DHA as a source of ω‐3 in rodents, showed increased glucose uptake (and increased translocation of GLUT‐4 to the membrane) by epididymal adipocytes from the group fed hipercaloric diet (Oh, Talukdar, Bae, Imamura, & Morinaga, 2011).

In addition to impaired metabolic functions, hypertrophied cells present an altered secretory pattern, resulting in increased secretion of pro‐inflammatory cytokines, and, according to the literature, reduction of anti‐inflammatory adipokines (Moraes‐vieira, Yore, Dwyer, Syed, & Aryal, 2015; Moreno‐Aliaga, Lorente‐Cebrián, & Martínez, 2010; O’Connell et al., 2010; Wronska & Kmiec, 2012). In our study, we tested the ability of isolated adipocytes from different adipose depots to secrete these cytokines in culture (for 30 hr) without the contribution or "noise" of macrophages. We observed a significant increase in the production of TNF‐α, IL‐6, resistin and IL‐10 by the adipocytes isolated from the ING and RP WAT and MCP‐1 by RP adipocytes as a consequence of the HF diet. This increase was completely reversed by the treatment with FO. Allaire et al. (2016) evaluated obese humans with low‐grade systemic inflammation for 9 weeks with EPA or DHA supplementation of 2.7 g/day, and showed that, in relation to the control group, EPA reduced IL‐6, LDL cholesterol, and TAG. However, with the exception of IL‐6, DHA further reduced LDL and TAG cholesterol, as well as CRP, IL‐18, TNF‐α, and total cholesterol, and increased adiponectin and HDL cholesterol. In another study evaluating the effect of EPA associated to a HF diet, EPA has been shown to improve inflammatory parameters by reducing MCP‐1 and macrophage recruitment. These authors evaluated the cellularity of gonadal adipose tissue and described that the group which received HF diet for 6 weeks and EPA for 5 weeks (added to the diet) presented no reduction of body mass, thus showing the effect of EPA independent of obesity (LeMieux et al., 2015). A reduction of adiponectin, and also of IL‐10 (which was not evidenced in our model), was expected since they are known as anti‐inflammatory cytokines. Sugama et al. (2017) analyzed the epididymal adipose tissue in the obese state or not, and suggested that the expression of pro‐ and anti‐inflammatory genes are increased together during WAT expansion in mice receiving HF diet, justifying that the balance of both cytokines is regulated by the supply of energy in the WAT, where the elevation of the anti‐inflammatory cytokines aims to balance the activities of the pro‐inflammatory ones. In this same work, they showed that intracellular ATP can regulate cytokine expression in epididymal WAT, and, in macrophage lineage, ATP has been positively correlated with TNFα and IL‐10 expression. Finally, our data challenge the exclusive relevance given to the immune system cells that infiltrate WAT under obesity conditions, further emphasizing the participation of adipocytes in metabolic homeostasis.

In summary, low food intake concomitant to high fat intake for 16 weeks, resulted in obesity, as well as related complications, such as insulin resistance, dyslipidemia, changes in mass, metabolism and secretory function of adipose tissue in both, the subcutaneous region as well as the visceral region. It was also observed a cellular hypertrophy, leading to loss of its original functions becoming more lipolytic and inflammatory. In turn, our results show that FO exhibits antiobesogenic effect as well as a potent action in the reversion of MS and adipocyte dysfunctions. It is noteworthy that these beneficial effects were observed in mice that remained fed a high‐fat diet. Although the benefits of omega‐3 FAs to the health has been widely reported, more studies are necessary to recommend the most effective dosages and formulas (as type of LC n‐3 PUFA, EPA/DHA ratio) for specific diseases. Herein, we showed that FO treatment 3 times per week, containing 5:1 EPA/DHA ratio, represent a potential therapeutic alternative to treat MS caused by the excessive intake of the HF diet. Our data also support that the depot‐specific effect of FO treatment on ING and RP adipocyte might be a relevant mechanism underlying all systemic effects.

## CONFLICT OF INTEREST

The authors declare that they have no competing interests.

## AUTHOR CONTRIBUTIONS

RS and MV designed the study and analyzed the data. MC, TF, VS, JS, and VA performed the animal study and acquired the data. RS, MT, and MV drafted and revised the manuscript. All authors read and approved the final manuscript.
